# Multi-target action of the novel anti-Alzheimer compound CHF5074: in vivo study of long term treatment in Tg2576 mice

**DOI:** 10.1186/1471-2202-14-44

**Published:** 2013-04-05

**Authors:** Sandra Sivilia, Luca Lorenzini, Alessandro Giuliani, Marco Gusciglio, Mercedes Fernandez, Vito Antonio Baldassarro, Chiara Mangano, Luca Ferraro, Vladimiro Pietrini, Maria Francesca Baroc, Arturo R Viscomi, Simone Ottonello, Gino Villetti, Bruno P Imbimbo, Laura Calzà, Luciana Giardino

**Affiliations:** 1 Department of Veterinary Medicine, University of Bologna, Bologna, Italy; 2 Health Science and Technologies Interdepartmental Center for Industrial Research (HST-ICIR), University of Bologna, Via Tolara di Sopra 50, Bologna, Ozzano Emilia I-40064, Italy; 3 IRET Foundation, Via Tolara di Sopra 50, Bologna, Ozzano Emilia 40064, Italy; 4 Department of Life Sciences and biotechnology, University of Ferrara, Via Fossato di Mortara 17–19, Ferrara, Italy; 5 Department of Neurosciences, Laboratory of Neuropathology, University of Parma, Via Gramsci 14, Parma 43100, Italy; 6 Department of Biosciences, Biochemistry and Molecular Biology Unit, Laboratory of Functional Genomics and Protein Engineering, University of Parma, Parma, Italy; 7 Research & Development, Chiesi Farmaceutici, Via Palermo 26/A, Parma 43100, Italy; 8 Present address: Microbiological Laboratory, GlaxoSmithKline Manufacturing Spa, Via Asolana 90, Parma, S.Polo di Torrile 43056, Italy

**Keywords:** Alzheimer’s disease, Cell-cycle events, CHF5074, Dendrite pathology, Tg2576 mice

## Abstract

**Background:**

Alzheimer disease is a multifactorial disorder characterized by the progressive deterioration of neuronal networks. The pathological hallmarks includes extracellular amyloid plaques and intraneuronal neurofibrillary tangles, but the primary cause is only partially understood. Thus, there is growing interest in developing agents that might target multiple mechanisms leading to neuronal degeneration. CHF5074 is a nonsteroidal anti-inflammatory derivative that has been shown to behave as a γ-secretase modulator *in vitro* and to inhibit plaque deposition and to reverse memory deficit *in vivo* in transgenic mouse models of Alzheimer’s disease (AD). In the present study, the effects of a long-term (13-month) treatment with CHF5074 on indicators of brain functionality and neurodegeneration in transgenic AD mice (Tg2576) have been assessed and compared with those induced by a prototypical γ-secretase inhibitor (DAPT).

**Results:**

To this end, plaque-free, 6-month-old Tg2576 mice and wild-type littermates were fed with a diet containing CHF5074 (125 and 375 ppm/day), DAPT (375 ppm/day) or vehicle for 13 months. The measured indicators included object recognition memory, amyloid burden, brain oligomeric and plasma Aβ levels, intraneuronal Aβ, dendritic spine density/morphology, neuronal cyclin A positivity and activated microglia. Tg2576 mice fed with standard diet displayed an impairment of recognition memory. This deficit was completely reverted by the higher dose of CHF5074, while no effects were observed in DAPT-treated mice. Similarly, amyloid plaque burden, microglia activation and aberrant cell cycle events were significantly affected by CHF5074, but not DAPT, treatment. Both CHF5074 and DAPT reduced intraneuronal Aβ content, also increasing Aβ40 and Aβ42 plasma levels.

**Conclusions:**

This comparative analysis revealed a profoundly diverse range of clinically relevant effects differentiating the multifunctional anti-inflammatory derivative CHF5074 from the γ-secretase inhibitor DAPT and highlighted unique mechanisms and potential targets that may be crucial for neuroprotection in mouse models of AD.

## Background

Extracellular amyloid plaques formed by aggregated β-amyloid (Aβ) peptides and intracellular neurofibrillary tangles are the landmarks of Alzheimer’s disease (AD) pathology [1]. Studies relying on familial AD patients with mutations in the Aβ (amyloid) precursor protein APP, or the catalytic subunits of the γ-secretase complex responsible for the second (Aβ-generating) step of APP processing yielded the so-called “β-amyloid cascade hypothesis” of AD. Recently, this etiopathogenetic hypothesis, which in its latest formulation holds Aβ oligomers as the most proximal cause of AD neurodegeneration [2], is facing the failure of multiple clinical trials carried out with various drugs targeting Aβ accumulation in the brain [3]. These include active and passive anti-Aβ vaccines, but also γ-secretase inhibitors and modulators, and Aβ aggregation inhibitors [4-7]. Additional abnormalities not so closely related to Aβ accumulation/overproduction *per se* (*e.g*. altered intraneuronal APP metabolism and trafficking) [8-10] and other derailed cellular processes whose precise causal relationship with Aβ remains to be elucidated, have been described in the AD brain. The multifaceted cellular dysregulation associated with AD neurodegeneration, possibly also leading to cortical atrophy, includes tau protein hyperphosphorylation, neuronal cell death, neuroinflammation, neurite abnormalities, synapse and dendritic spine loss [10-16]. Thus, the search for new (and effective) AD therapies needs to address additional targets other than just amyloid clearance and to focus on early prevention of neurodegeneration.

CHF5074 [1-(3′,4′-dichloro-2-fluoro[1,1′-biphenyl]-4-yl)-cyclopropanecarboxylic acid] is a nonsteroidal anti-inflammatory derivative devoid of cyclooxygenase inhibitory activity. *In vitro*, CHF5074 behaves as a γ secretase modulator preferentially inhibiting Aβ42 production, without affecting neither the processing of the C-terminal portion of APP, nor γ-secretase-mediated Notch processing [17-19]. CHF5074 has been shown to inhibit brain plaque deposition and to attenuate or reverse contextual and spatial memory deficit in different transgenic mouse models of AD, also reversing long-term potentiation deficit in the hippocampus [20-22]. However, recent data have shown that CHF5074 is able to promote axon growth and astrocyte plasticity by modulating Rho-GTPase-dependent signaling [23], thus suggesting that the range of biological actions exerted by this compound may be wider than expected. In the present *in vivo* and *ex-vivo* study, we evaluated the effect of a long-term treatment with CHF5074 on cognitive performance and on several neuronal dysfunction markers in the Tg2576 mouse model of AD. The effects of CHF5074 were compared with those of DAPT (N-[N-(3,5-difluorophenacetyl)-L-alanyl]-S-phenylglycine t-butyl ester), a prototypical γ-secretase inhibitor [24]. Drug treatments were started at 6 months of age when no plaques are detectable [25], and were carried on in a chronic fashion till a very late age (19 months), which is close to the life expectancy of Tg2576 mice. A recognition memory test was carried out before the analysis of several distinct post-mortem indicators of brain functionality and neurodegeneration.

## Methods

### Animals and treatments

Tg2576 transgenic mice carry a transgene coding for the 695-amino acid isoform of human APP derived from a large Swedish family with early-onset AD [25]. These mice express high concentrations of the mutant Aβ, develop significant number of amyloid plaques and display memory deficits. Tg2576 mice and their non-transgenic littermates (001349-W), which served as controls, were purchased from Taconic Europe (Lille Skensved, Denmark). Mice were all genotyped for retinal degeneration.

Six-months-old transgenic females and aged-matched non-transgenic littermates were used. Transgenic and non-transgenic mice (N = 17–27 per treatment group) were treated for 13 months with CHF5074 (125 and 375 ppm in the diet), DAPT (375 ppm/day in the diet) or standard diet (herafter designated as “vehicle”). CHF5074- and DAPT-medicated diets were provided by Mucedola (Settimo Milanese, Italy). The estimated ingested doses of CHF5074 were about 20 and 60 mg/kg/day (behaviourally effective dose when given chronically for 9 months) [21]; the estimated ingested dose of DAPT was about 60 mg/kg/day. The dose of DAPT was selected based on previous studies showing that the drug given orally at doses ranging from 10 to 100 mg/kg dose-dependently inhibited cortical Aβ levels in hAPP transgenic mice [26]. After behavioural testing, mice were sacrificed and their brains were split in the two hemispheres. The left hemisphere was divided into anterior and posterior parts at level −0.70 according to the Paxinos and Franklin atlas of the mouse brain [27]. The anterior part, containing the olfactory bulb, was quickly frozen into liquid N2, while the posterior part, including the hippocampus, was fixed for amyloid plaque and Aβ oligomer analysis. The right hemisphere was divided as above and fixed for immunohistochemistry (anterior part) and Golgi-Cox staining (posterior part, including the hippocampus). Animal care and treatments were in accordance with the EU Directive 2010/63/EU for animal experiments and in conformity with protocols approved by the Ethical Committee of Animal Experimentation, University of Bologna.

### Novel object recognition test (NOR)

Long-term memory was evaluated in 18 months-old mice using NOR, measuring recognition memory under spontaneous behavioural conditions. Before NOR, all animals were tested for the papillary reflex and only positive animals were included in the study. Mice were tested in an open-square grey arena (46 x 46 cm), 30 cm high (Ugo Basile, Comerio, Italy). The task started with a habituation trial in which the animals were placed into the empty arena for 10 min. The next day, mice were placed into the same arena containing two identical objects (familiarization phase). In order to evidence side preferences, exploring times spent on left and right familiar objects were recorded separately. The exploratory behaviour was analyzed by calculating the investigation time on both objects. Sniffing and touching the object at a distance not greater than 2 cm were scored as object investigation. Four hours later (test trial) mice were placed in the arena containing one object identical to the one presented during the familiarization phase (familiar object), and a new one (novel object); the time spent exploring the two objects was recorded for 10 min. The videotracking software AnyMaze (Stoelting, Wood Dale, Illinois) was used for analysis. Memory was expressed as recognition index: (seconds on novel – seconds on familiar)/(total time on objects). Animals with no memory impairment spent longer time investigating the novel object, giving a higher discrimination index.

### Golgi-Cox staining

FD Rapid GolgiStain™ Kit (FD NeuroTechnologies, Ellicott City, MD, USA) was used for detailed morphological analysis of dendritic spines. Tissue was rapidly frozen, cut on a cryostat and mounted on gelatin-coated microscope slides. Sections were processed with the NDT104 FD Rapid GolgiStain™ Kit. For each mouse, ten fully impregnated hippocampal CA1 neurons were identified under low magnification (20×, 0.5NA). The acceptance criteria for spines were according to Middei et al. [28]. Dendritic spines were measured under high magnification (100×, 1.25NA) from images acquired using a digital camera (Olympus F-View). Measurements were performed on secondary and tertiary branches of CA1 basal dendrites irrespective of their orientation. On each neuron, at least five dendritic spines were selected according to the criteria proposed by Knafo et al. [29]: only considering spines separated throughout their entire length from neighbour spines or dendrites and excluding spine-like protrusions with bifurcated heads or with heads longer than 3.5 μm. Spine length was defined as the distance from the dendritic shaft to the tip of the spine.

### Immunohistochemistry and image analysis

Indirect immunofluorescence was used to determine immunoreactivity associated with intracellular Aβ, cyclin A, NeuN. Brain tissue was immersed in 4% paraformaldehyde for 24 hours and then washed in 5% sucrose in phosphate buffer. Sections (14 μm thick) were cut with a cryostat to include the medial cortex. The following primary antibodies were used: 6E10 anti-Aβ_1–16_ monoclonal antibody (Covance, Princeton, NJ) for APP/Aβ (this antibody reacts with the sAPPβ precursor as well as with the processed forms of Aβ) at a 1:1000 dilution; 4G8 anti-Aβ_17–24_ monoclonal antibody (Covance) for APP/Aβ (this antibody reacts with the sAPPβ precursor as well as with the processed forms of Aβ) at a 1:800 dilution; rabbit anti Cyclin A (ab7956, AbCam, Cambridge, UK) diluted 1:200; mouse anti neuronal nuclei antibody (NeuN, MAB377 Chemicon) diluted 1:200. Appropriate Rhodamine Red-X-and FITC-conjugated secondary antibodies were used for detection (Jackson ImmunoResearch, Baltimore, PA).

For intraneuronal Aβ immunostaining, about 50 neurons in the anterior cingulated cortex were analyzed for each animal. Stained specimens were analyzed with a Nikon 600 Eclipse microscope equipped with a Nikon DXM1200F digital camera (Nikon Italia, Florence, Italy). The ProPlus software (Media Cybernetics Inc, Bethesda, MD) was used to evaluate optical density in single cells. The expression of cell cycle proteins was analyzed in two sections/animal, in both genotypes. NeuN-positive cells were scored within layers II/III of the frontal cortex, and the percentage of cyclinA immunoreactive, NeuN-positive cells was recorded [30]. For all markers, the mean value/animal was used for statistical analysis. All analyses were performed in a blinded manner.

### Brain β-amyloid plaques and activated microglia

Brain samples corresponding to the posterior half of the left hemisphere were used for quantitative analysis of plaques and activated microglia. They were fixed in 10% formalin and then embedded in paraffin according to a standard procedure. Coronal sections (10 μm thick) ranged from bregma −1.46 mm (anterior) to −2.06 mm (posterior) [27]. Aβ plaque immunohistochemistry was performed using the biotinylated 6E10 monoclonal antibody (Signet Laboratories, Dedham, MA) diluted 1.250 as primary antibody. Pretreatments were: incubation in a 3% H_2_O_2_ solution in distilled water for 15 minutes to block endogenous peroxidase; incubation in 80% formic acid for 30 minutes for antigen retrieval. After rinsing in TBS for 10 minutes, sections were incubated overnight at 4°C in a humid atmosphere with the primary antibody diluted in TBS containing 0.3% Triton X-100. After rinsing in TBS for 10 min, sections were incubated for 60 min in a humid atmosphere with the streptavidin-peroxidase solution, according to the mouse-on-mouse kit procedure (Dako Cytomation, Glostrup, Denmark) using a peroxidase-based revealing system. Peroxidase activity was detected by treatment with 3,3′-diaminobenzidine (DAB) for 5 minutes. Slides were photographed using a digital Nikon DS microscope colour camera. Digital images were analyzed using NIS-Elements software (Nikon, Tokyo, Japan). Each image was analyzed using the automated target detection mode. Imagesize was 1280 x 960 pixels with a target area size of 68,000 μm^2^. The software determined the number of plaques, the plaque mean area and the plaque area fraction (immunopositive area/total area used as scan object). Twelve counts were performed for each of the two levels considered. Analyses were performed in analogous areas of the cortex and hippocampus using a 10x objective.

Activated microglia in CA1 region of hippocampus was immunodetected using the Iba1 Rabbit polyclonal antibody (Biocare, Concord, CA). A 20x objective and a target area of 16,900 μm^2^ was used for this analysis, which involved three counts in homologous areas of CA1 region of hippocampus. After rinsing in distilled water, sections were incubated in a 3% H_2_O_2_ solution in distilled water for 15 minutes. After rinsing in TBS for 10 minutes, sections were incubated for 30 minutes with normal goat serum diluted 1:20 with TBS. The sections were then incubated overnight at 4°C in a humid atmosphere with the primary Iba1 antibody diluted 1:250 with TBS. After rinsing in TBS for 10 min, sections were incubated for 60 min in a humid atmosphere with the secondary antibody solution provided by the Goat anti Rabbit Envision System (Dako, Denmark) diluted 1:1 with TBS. Peroxidase activity was detected by treatment with DAB.

### Brain Aβ oligomers

Aβ oligomers were determined in low-detergent (0.1% Triton-X100, 0.01% Nonidet-P40) and high-detergent (3% SDS, 0.5% Triton X-100, 1% deoxycholate) extracts prepared from left hemi-forebrains specimens as described by Lesné et al. [31] with slight modifications. These included cumulative extraction (50 mM Tris–HCl, pH 7.6, 150 mM NaCl, 2 mM EDTA, 0.01% NP40, 0.1% Triton X-100, plus 1 mM phenylmethylsulfonyl fluoride) and mixing of the soluble (extracellular-enriched) and cytoplasmic fractions, and the omission of the endogenous immunoglobulin depletion step in order to avoid any artefactual modification of the samples. The residual pellet remaining after low-detergent extraction was further extracted by gentle agitation in 200 μl of a buffer solution containing 50 mM Tris–HCl (pH 7.4), 150 mM NaCl, 0.5% Triton X-100, 1 mM EDTA, 3% SDS, 1% deoxycholate and 1 mM PMSF, followed by centrifugation for 90 min at 13,000 rpm. Total protein content of each extract was determined in triplicate with the Bradford Protein Assay reagent (Bio-Rad) using bovine serum albumin as standard; the average of the resulting values (differing by less than 5% of the mean) was used to balance sample input for immunoblot analysis. All extractions were performed in three experimental sessions, carried out at three-days intervals on randomized subsets of the various brain samples, and the resulting extracts (a total of 65) were flash-frozen in dry ice and stored at −80°C. For immunoelectrophoretic analysis, brain extracts (100 μg or 50 μg total protein/each for the low-detergent and the high-detergent extracts, respectively) were heated for 15 min at 70°C in sample buffer and electrophoresed on pre-cast 4-12% Bis-Tris Midi gradient gels (Invitrogen). Gel-fractionated proteins were electro-transferred to 0.2 μm nitrocellulose membranes, which were boiled for 25 sec in PBS, soaked for 10 min in PBS containing 100 mM β-mercaptoethanol and 0.1% SDS, washed three times (10 min each) with PBS and blocked with 5% bovine serum albumin in Tris-buffered saline, prior to antibody addition and incubation using the Snap i.d. blotting system (Millipore). The 6E10 monoclonal antibody (diluted 1:500) was used for immunodetection. Antigen-antibody complexes were revealed with a goat anti-mouse, IrDye 680-labeled secondary antibody (LI-COR; diluted 1:3000), followed by visualization and quantification of immune-reactive bands by near-infrared fluorescence with an Odyssey imager (LI-COR). A total of 14 independent sets of analyses, each comprising 10 sample extracts with at least one vehicle-treated wild-type control, were performed. Non-specific, 6E10 mAb cross-reactive polypeptides, present in both wild-type and Tg2576 brain extracts, were used as loading controls and internal references for data normalization. Synthetic prefibrillar Aβ42(n) prepared according to Lambert et al. [32], with *n*-values ranging from 1 to 4, was used as size standard for electrophoretic analysis. Results, expressed as NIRF arbitrary units, were analyzed with the SigmaStat™ software and are presented as mean ± standard error of the mean (SEM).

### Aβ40 and Aβ42 plasma levels

At the time of sacrifice, blood samples were collected in EDTA-vacuum collection tubes, centrifuged at 4000 *g* for 10 min and plasma aliquots were stored at −80°C. Simultaneous quantification of β-amyloid_1-40_ (Aβ40) and β-amyloid_1-42_ (Aβ42) peptides was performed on properly diluted plasma samples using the INNO-BIA plasma Aß forms kit (Innogenetics NV, Gent, Belgium, through Innogenetics srl, Italy), following the manufacturer’s instructions. This kit is a fluorimetric bead-based immunoassay using Luminex xMAP® technology. Briefly, the Aβ40 and Aβ42 peptides are captured selectively by a mix of beads (xMAP® microspheres) coated with three monoclonal antibodies (mAb): 21 F12 for Aβ42, 2G3 for Aβ40 and AT120 for matrix. Following an overnight incubation step at 4°C, the mix is washed and subsequently incubated with the “detection conjugate” solution (phycoerythrin-labeled streptavidin) at room temperature for 1 h. The mix of beads is then washed and read using the Luminex® 100™ IS Total System, which analyzes the microspheres/beads in a flow stream. The fluorescence signals associated with individual beads are converted into intensity units by a digital signal processor and then related to the concentration of the bound antigen through the Luminex IS 2.3 software.

### Statistical analysis

Data were analyzed with the appropriate model of analysis of variance (ANOVA) depending on the type of variable. Behavioural data were analyzed with two-way analysis of variance with “genotype” (non-transgenic and Tg2576) and “treatment” (vehicle, CHF5074/DAPT) as fixed factors and mouse as random factor. For balanced design (object recognition task), the ANOVA model also included “genotype by treatment” as fixed factor. To reduce the loss of power due to multiple testing post hoc comparisons were only directed toward the transgenic control group (Tg2576-vehicle) and were carried out with the Holm-Sidak’s test. Two-tailed *p* values were calculated. Calculations were performed with the statistical software SigmaStat™ (Version 3.5, SPSS, Chicago, IL). Results were generally presented as mean ± standard error of the mean (SEM).

## Results

### Drug tolerability

**Figure 1:**
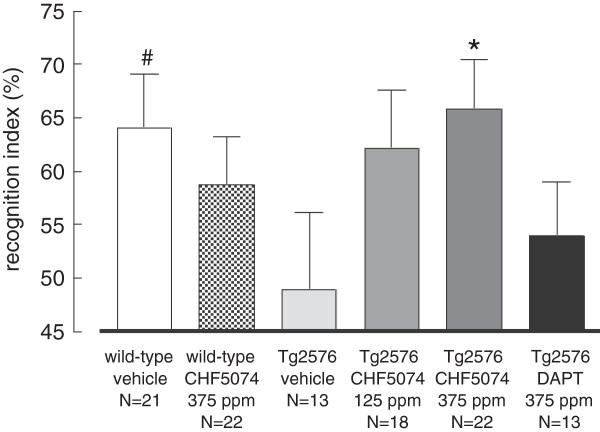
**Novel object recognition memory in the different treatment groups.** Bars represent the average (± SEM) of the recognition index in the novel object recognition task. Vehicle-treated Tg2576 mice showed a borderline significant impairment of recognition compared to control non-transgenic mice treated with vehicle (*p* = 0.052). CHF5074-treatment (375 ppm) completely reversed memory impairment in Tg2576 mice (*p* = 0.031). DAPT was ineffective both in Tg2576 and wild-type animals *(wild type not shown).* The number of animals included in each group is indicated under the corresponding bar. *p < 0.05 and # *p* = 0.052 vs vehicle-treated Tg2576 mice.

Total mortality during the 13 months-time period of the present study was 28% and 15% for Tg2576 mice and wild-type animals, respectively (Figure 1). Mortalities in vehicle-, CHF5074 (375 ppm/day)- and DAPT (375 ppm/day)-treated wild-type animals were 15%, 15% and 30%, respectively. The corresponding values for vehicle-, CHF5074 (125 ppm/day)-, CHF5074 (375 ppm/day)- and DAPT (375 ppm/day)-treated Tg2576 mice were 29%, 28%, 28% and 47%, respectively. Mortality was not statistically different among treatment groups. Overall, CHF5074 and DAPT, continuously administered for 13 months, appeared to be well tolerated by both wild-type and Tg2576 animals.

### Object recognition memory performance of 18-months-old Tg2576 mice chronically treated with CHF5074 or DAPT

Object recognition memory, measured by the novel object recognition (NOR) task after 12 months of drug treatment, was evaluated as a first indicator of CHF5074 efficacy. As shown in Figure 1, compared to wild-type animals, Tg2576 mice fed with standard diet (“vehicle”) displayed a borderline significant impairment of recognition memory (*p* = 0.052). This impairment was fully recovered in transgenic animals chronically treated with the higher dose of CHF5074 (375 ppm/day; *p* = 0.031), while it was not significantly modified by the lower dose of CHF5074 (125 ppm/day; *p* = 0.101). CHF5074 treatment did not affect recognition memory in wild-type mice. Chronic treatment with DAPT (375 ppm/day) did not affect the NOR performance of either wild-type (data not shown) or Tg2576 mice. Exploration time among the experimental groups was not different in the training period (one way ANOVA, p = *p* = 0.365).

To exclude possible alterations of either motor or exploratory behaviour as possible influencing factors, the mean speed re\corded during the 10 min habituation trial in the empty arena, and the total object exploration time during the familiarization phase were also analyzed. Tg2576 mice treated with both doses of CHF5074 displayed a significant (*p* < 0.01) reduction in the mean speed when compared to vehicle-treated mice (3.42 ± 0.64, 2.13 ± 0.24 and 2.19 ± 0.23 m/min after vehicle, CHF5074 125 and CHF5074 375 ppm/day, respectively). However, this effect was not associated with significant differences in total time in movement (494 ± 26, 474 ± 28 and 511 ± 15 sec after vehicle, CHF5074 125 and 375 ppm/day, respectively). Neither CHF5074 nor DAPT significantly affected the mean speed (1.88 ± 0.18, 2.31 ± 0.18 and 1.72 ± 0.18 m/min after vehicle, CHF5074 375 ppm/day and DAPT 375 ppm/day, respectively) or the total time in movement (470 ± 19, 514 ± 14 and 459 ± 20 sec after vehicle, CHF5074 375 ppm/day and DAPT 375 ppm/day, respectively) of wild-type animals.

### Amyloid plaque burden, intraneuronal Aβ and Aβ clearance in the brain of 18-months-old Tg2576 mice chronically treated with CHF5074 or DAPT

**Figure 2:**
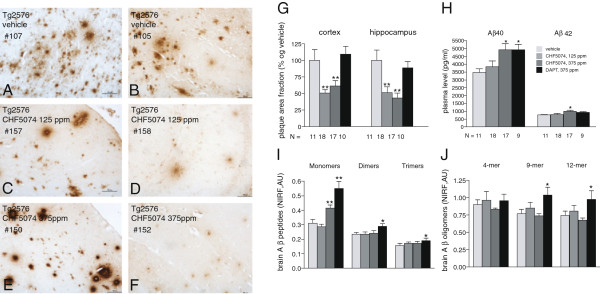
**Effect of CHF5074 and DAPT treatments on Aβ-related, cellular and molecular parameters.** Representative images of amyloid plaques stained with the 6E10 monoclonal antibody in the cerebral cortex (**A**, **C**, **E**) and in the hippocampus (**B**, **D**, **F**) of untreated (vehicle, **A**, **B**) and CHF5074-treated Tg2576 mice (**C**-**F**) are shown in the figure. CHF5074 dose and animal code number (#) are indicated in each panel. Quantification of plaque immunostaining in both brain areas of untreated (“*vehicle*”), CHF5074-treated and DAPT-treated animals (indicated by the different bar colours) is shown in panel **G**. Plasma Aβ40 and Aβ42 levels in the same groups of untreated, CHF5074- and DAPT-treated animals are reported in panel **H**. The levels of monomeric Aβ and of different Aβ oligomeric species detected with the 6E10 antibody in low-detergent (0.1% Triton-X100, 0.01% Nonidet-P40) and high-detergent (3% SDS, 0.5% Triton X-100, 1% deoxycholate) brain extracts are shown in panels **I** and **J**, respectively. Data refer to the quantification of the indicated Tg2576-specific immunoreactive bands (i.e., polypeptides not present in brain extracts from wild-type littermates) in untreated Tg2576 mice and in Tg2576 mice treated with CHF5074 or DAPT (same number of animals/group as in panel **G**) by near-infrared fluorescence (NIRF; arbitrary units). Data in panels **G**-**J** are expressed as mean ± SEM; **p < 0.01 or *p < 0.05 vs vehicle-treated Tg2576 mice; the number of animals in each group is indicated below the corresponding bar (see Additional file 2: Figure S1 for a representative example of gel fractionation and immunoblot analysis).

Amyloid plaque burden was visualized with the anti-Aβ_1–16_ monoclonal antibody (mAb) 6E10 (Figure 2A-F; Additional file 1: Figure S2) and was semi-quantitatively determined by digital image analysis of coronal brain sections derived from the cortex and the hippocampus (Figure 2G). CHF5074 treatment modified both the amount and the distribution of amyloid plaques. Despite a higher plaque abundance in the cortex compared to the hippocampus, at either dose it caused a nearly 50% reduction of the plaque area fraction in both brain regions. Quantification of the immunoreactivity revealed a significantly lowered plaque area fraction in CHF5074-treated animals compared to control mice, both in the cortex (−61.0 ± 5.1%, p < 0.001 and −48.2 ± 8.9%, p = 0.003 in the 125 and 375 ppm/day-treated groups, respectively) and in the hippocampus (−52.3 ± 9.4%, p = 0.007 and −64.9 ± 8.1%, p = 0.001 in the 125 and 375 ppm/day-treated groups, respectively). DAPT treatment did not affect plaque burden.

In contrast, as further shown in Figure 2 (panels I, J), no significant effect of CHF5074 treatment was observed on brain Aβ oligomers (see Additional file 2: Figure S1 for a representative example of gel fractionation and immunoblot analysis). The only significant change revealed by this analysis was an increase of brain monomeric Aβ levels in Tg2576 mice treated with the higher dose of CHF5074, which was associated with increased plasma levels of both Aβ40 and Aβ42 (Figure 2H). An even higher increase of brain Aβ monomer levels was observed upon treatment with DAPT, which also caused a significant elevation of Aβ oligomeric species migrating as dimers, trimers and nonamers, and a slight (borderline significant) increase of 12-mers (Figure 2I, J).

**Figure 3:**
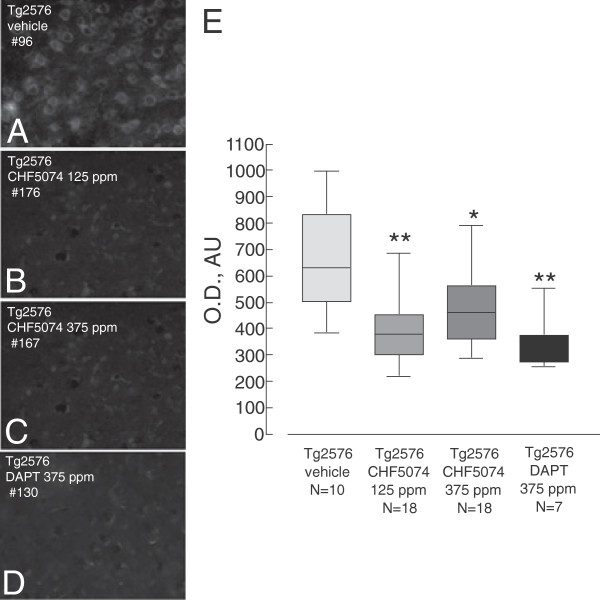
**Effect of CHF5074 and DAPT treatments on 6E10 intraneuronal immunoreactivity. A**-**C**. Representative images of 6E10-immunostaining of intraneuronal 6E10-immunoreactivity in cerebral cortex sections of vehicle- (**A**), CHF5074 125 ppm- (**B**), CHF5074 375 ppm- (**C**) and DAPT 375 ppm-treated Tg2576 mice (**D**); # indicates the animal code. **E**. Quantification of intraneuronal 6E10-immunoreactivity in the different treatment groups. The number of animals included in each group (N) is indicated under the corresponding bar, data are expressed as mean ± SEM, *p < 0.05 and **p < 0.01 for CHF5074 treated vs untreated Tg2576 mice.

Intraneuronal Aβ (APP) levels were assessed using mAb 6E10 and 4G8 as detection reagent. Representative images of brain cortex specimens stained using mAb 6E10 derived from untreated (panel A; “vehicle”) and CHF5074-treated (panels B, C; 125 and 375 ppm) mice are shown in Figure 3. A paper by Aho et al. [33] directly compared all commercially available monoclonal antibodies in post-mortem Alzheimer brain tissues, concluding that intracellular labeling was readily apparent when using 6E10 and that both 6E10 and 4G8 antibodies failed to distinguish Aβ peptides from APP. According to these findings, we will use the term “6E10-immunoreactivity” to describe the results obtained with this antibody. Mean (background subtracted) optical density values derived from at least 50 neurons/animal sampled in the anterior cingulated cortex were used for statistical analysis. As revealed by this analysis (Figure 3D), 6E10-immunoreactivity was significantly lower in Tg2576 mice treated with either dose of CHF5074 (−33.5 ± 7.3%, p < 0.01; -27.1 ± 4.9%, p < 0.01) or DAPT (−44 + 10.6, p < 0.01) than in vehicle-treated animals. The same results were obtained using 4G8 antibody *(data not shown).*

### Anti-neuroinflammatory properties and possible downstream effects of chronic treatment with CHF5074 or DAPT

#### Microglia activation

**Figure 4:**
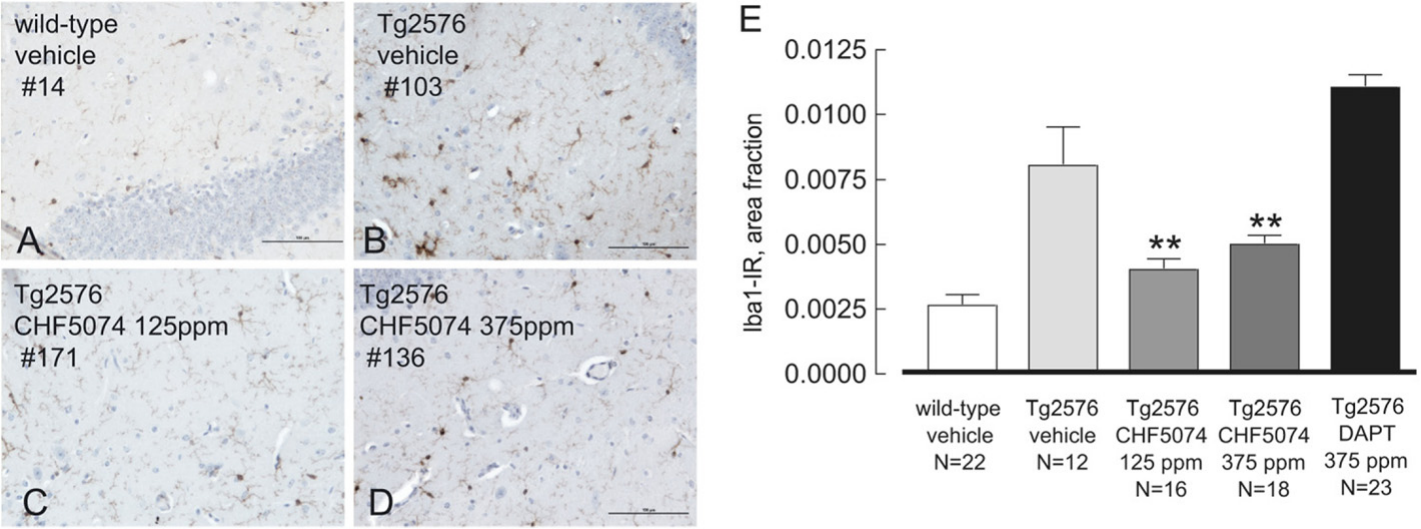
**Effect of CHF5074 and DAPT treatments on microglia activation. A-D**. Representative images of Iba1-immunostaining of activated microglia in hippocampus sections from wild-type (**A**) and Tg2576 vehicle- (**B**) and CHF5074-(**C**, **D**)-treated animals; # indicates the animal code. **E**. Quantification of Iba1-immunostaining of activated microglia in the different treatment groups. CHF5074, but not DAPT, reduces the level of microglial activation observed in Tg2576 mice. **p < 0.01 vs vehicle-treated Tg2576 mice.

Microglia activation was investigated with the use of an antibody targeting the glia-specific calcium-binding adaptor protein Iba1. Representative images of the hippocampal CA1 region from vehicle- and CHF5074-treated transgenic mice are shown in Figure 4 (panels A-D; Additional file 1: Figure S2). Activated, immunopositive microglia was lower (−66.3 ± 5.3% *p* < 0.001) in wild-type animals than in control, vehicle-treated Tg2576 mice. Interestingly, compared to Tg2576 control mice, activated microglia in the hippocampus of CHF5074 (125 and 375 ppm/day)-treated Tg2576 mice was significantly (*p* < 0.001) reduced (−50.2 ± 5.2% and −45.5 ± 4.5%, respectively). On the contrary, DAPT treatment did not affect activated microglia in Tg2576 mice (+ 19.3 ± 18.0% increase).

#### Dendritic spine length and density

**Figure 5:**
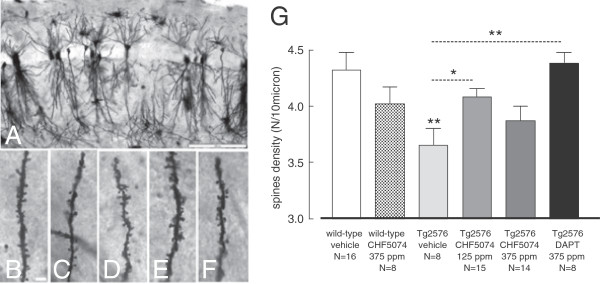
**Effect of CHF5074 and DAPT treatments on dendritic spine density and length in hippocampal pyramidal neurons.** Low-power mag of CA1 hippocampal cortex (**A**) visualized by the Golgi-Cox technique; **B**-**E**: high-power mag (100x, 1.35NA) of spine morphology in hippocampal pyramidal neurons of wild-type (B), Tg2576 vehicle- (**C**), Tg2576 CHF5074 125 ppm- (**D**), CHF5074 375 ppm- (**E**) and DAPT 375 ppm- (**F**) treated mice. G. Quantification of the data, including those related to DAPT treatment, expressed as mean (± SEM) spine density and length of pyramidal neurons from the cerebral cortex of animals belonging to different experimental groups; the number of animals utilized for this analysis is indicated below the bars corresponding to each treatment group. *p < 0.05; **p < 0.01. Bars: A, 200 μm; B, 5 μm.

Dendritic spine length and density were next evaluated in hippocampal pyramidal neurons of the CA1/CA2 fields by GolgiCox silver impregnation. As shown in Figure 5, a reduction of spine density compared to age-matched wild-type animals (*p* < 0.001) was apparent in the hippocampus of vehicle-treated, control Tg2576 mice. In contrast, no significant difference in hippocampal spine length was observed between wild-type and transgenic animals (*data not shown*). While DAPT fully prevented dendritic spine density reduction in Tg2576 mice, a clear and statistically significant amelioration effect of CHF5074 (*p* = 0.046 compared to vehicle-treated transgenic animals) was only observed at the lower dose (125 ppm/day) of the compound.

#### Cell cycle functionality

**Figure 6:**
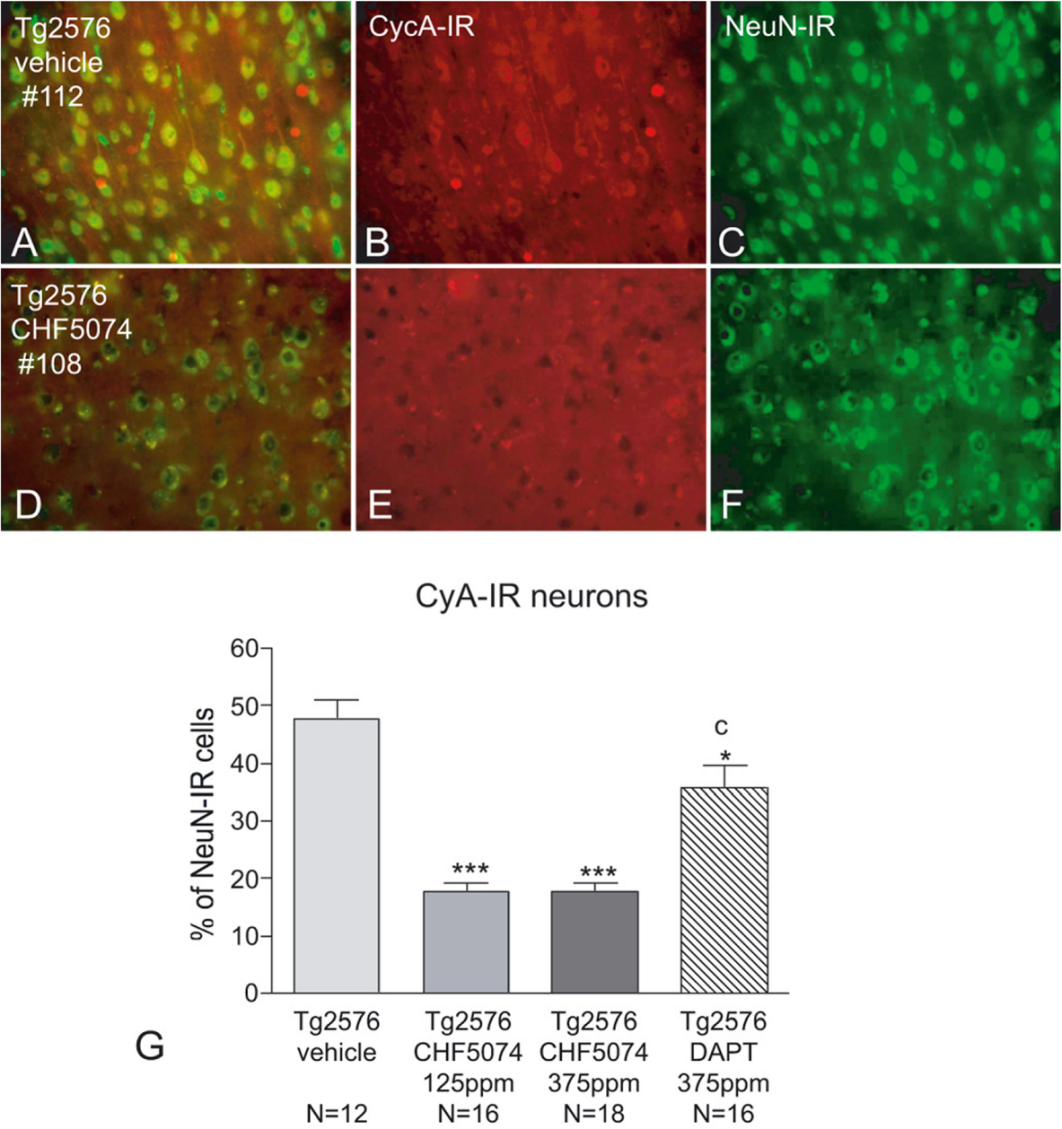
**Effect of CHF5074 and DAPT treatments on neuronal cell-cycle functionality. A**-**F**. Representative images of cyclin A immunoreactivity (red staining, **B** and **E**) in NeuN-positive cells (green staining, **C** and **F**) and of the resulting merge images (**A**, **D**) in Tg2576-mice treated with vehicle (**A**-**C**) or with CHF5074 375 ppm (**D**-**F**); # indicates the animal code. **G**. Quantification of cyclin-A positive cells versus total NeuN-positive cells in the different treatment groups. While approximately 50% of neurons express Cyclin A in untreated (“*vehicle*”) Tg2576 mice, the percentage of Cyclin A/NeuN-positive neurons is reduced to less than 20% in CHF5074-treated, but not DAPT-treated, animals. * *p* < 0.05 and ***p < 0.001 vs vehicle-treated Tg2576 mice; c *p* < 0.001 vs CHF5074 125 and 375 ppm-treated Tg2576 mice.

Cell cycle functionality was then investigated in mature neurons by monitoring the levels of a regulatory molecule such as cyclin A (CycA), detected by immunostaining of neuronal nuclei (NeuN). Representative images from vehicle (A-C) and CHF5074-treated Tg2576 mice (D-F) are reported in Figure 6, where panels B and E refer to CycA, panels C and F to NeuN and panels A and D to the merged images. The fraction of CycA-positive cells over the total number of NeuN-positive neurons was counted in layers II-III of the medial cerebral cortex. In wild-type and Tg2576 control mice the percentage of CycA-expressing neurons was 8.6 ± 1.5% and 47.8 ± 3.3%, respectively. As shown in Figure 6G, at both 125 and 375 ppm/day doses, CHF5074 reduced the percentage of CycA-positive neurons (17.6 ± 1.5% and 17.8 ± 1.3%, respectively; *p* < 0.001 vs vehicle-treated control Tg2576 mice). DAPT treatment was less effective than CHF5074 in reducing the percentage of CycA-positive neurons (p < 0.05), leaving an average 35% of neurons still positive to CycA immunostaining (Figure 6, panel G).

## Discussion

Age is a major risk factor for AD and various pathophysiological changes, not all directly related to Aβ accumulation, take place in the ageing brain. Thus, when assessing the preclinical therapeutic efficacy of a candidate AD modifying drug, it is of the utmost importance that multiple indicators of brain functionality are examined in animal models of AD at the latest possible age. Furthermore, as most AD-associated changes tend to be irreversible, treatment should be started at a relatively early age, when no (or very few) symptoms of disease are detectable, and carried on in a chronic fashion till very late age (*i.e*. under conditions in which drug tolerability and lack of detrimental side-effects may become a major concern). Based on this background, in the present study the effects of chronic treatments with the nonsteroidal anti-inflammatory γ-secretase modulating compound CHF5074 or the γ-secretase blocker compound DAPT on cognitive performance as well as on several distinct post-mortem indicators of brain functionality and neurodegeneration have been compared in a transgenic mouse model of AD.

The present findings indicate that CHF5074, continuously administered for 13 months at 125 and 375 ppm/day doses, appears to be well tolerated by both wild-type and Tg2576 animals. In line with this observation, CHF5074, which is under development for the prevention and treatment of AD, displayed a safe and well tolerated profile, as established in an ascending dose regimens, double-blind, placebo-controlled, parallel group study [34]. At marked variance with γ-secretase blockers, here typified by DAPT (but see also e.g., [35]), no trends towards deleterious effects across different dosing cohorts on both “paper and pencil” and computerized cognitive tests have been described so far for CHF5074 [36].

Previous studies indicated that CHF5074 treatment may attenuate or reverse contextual memory deficit when given acutely [22] or chronically (8-month treatment) [21] to young Tg2576 mice. A reduction of spatial memory deficit was similarly observed following chronic treatment in a different mouse model of AD (TADS-41) [19]. A reversal of object recognition memory as well as hippocampal plasticity deficits was also observed upon CHF5074 subchronic treatment of young Tg2576 mice [22]. In this study we extended these observations by demonstrating the efficacy of a further prolonged (13 months) treatment with CHF5074 (375 ppm/day) in reversing object recognition memory deficit in Tg2576 mice at a very old age. This test, which is based on spontaneous animal behavior and does not require any exogenously applied stress, is widely used for memory evaluation in mouse models of AD [37-39]. To our knowledge, no previous study has evaluated the potential efficacy of a candidate AD drug in preventing cognitive deficit at such a late stage of animal life. This finding opens up the possibility that CHF5074 could be beneficial in reducing cognitive impairment also in late AD provided that treatment is started relatively early in the course of illness. Under the same experimental conditions, DAPT was ineffective in improving visual memory performance. This finding is in line with the previously observed failure of γ-secretase inhibition to rescue object recognition deficits in APP-overexpressing mice [40] and suggests that some cellular and molecular parameters are differentially affected by CHF5074 and DAPT. This view is further supported by the differential responses of some main pathology markers observed in Tg2576 mice treated with CHF5074 or DAPT (summarized in Table 1).

**Table 1:** Comparison between the main pathology markers in Tg2576 mice chronically treated either with a γ-secretase inhibitor (DAPT) and a mixed γ-secretase modulator with anti-inflammatory properties (CHF5074)

**Task/marker**	**DAPT**	**CHF5074**
Novel object recognition test *(recognition index)*	unchanged	restored at wild-type level at 375ppm
amyloid plaque burden	unchanged	reduced
oligomers		
plasma Aβ42	increased	increased at 375ppm
intraneuronal Aβ	reduced	reduced
microglia activation *(Iba1-IR)*	unchanged	reduced
Dendritic spine density *(CA1/2 pyramidal neurons)*	restored at wild-type level	increased at 375ppm, compared to Tg2576 vehicle
Aberrant cell cycle events *(cyclinA-IR neurons)*	lightly reduced (−28%)	strongly reduced (−63%)

Treatments were started in 6month old rats and protracted for 13 months. Behavioral test was then carried out in 18month old animals, and all post-mortem observation in 19month old animals.

Four of the biochemical indicators analyzed in the present study directly bear on Aβ and its brain accumulation/mobilization. These include amyloid burden, brain Aβ oligomers, plasma Aβ and intraneuronal APP/Aβ levels. Amyloid burden was markedly reduced in CHF5074-, but not in DAPT-treated animals. On the contrary, intraneuronal APP/Aβ and plasma levels of Aβ40 and Aβ42 were similarly modified by both treatments. The increase of plasma Aβ levels observed after chronic treatment with DAPT appears paradoxical. Although the effects of chronic treatment with DAPT on plasma or brain Aβ levels are not known, it has been observed that treatment of transgenic mice and humans with other gamma-secretase inhibitors (e.g., semagacestat) may cause late rebound effects on plasma Aβ levels [41]. Both CHF5074 and DAPT did not affect Aβ oligomers, either in high-detergent (membrane/intracellularly enriched) or in low-detergent (extracellularly enriched) brain extracts. In the case of CHF5074, the only observed effect was a small, but statistically significant increase in brain Aβ monomers. This effect was magnified in Tg2576 mice treated with DAPT, which also caused a statistically significant increase of some Aβ oligomeric species. Although the reason for this quite unexpected finding is presently unclear, it is conceivable to imagine that given the extremely prolonged treatment duration time this may reflect the “Aβ rise” phenomenon previously observed with DAPT and other γ-secretase inhibitors in the presence of suboptimal inhibitor and/or CTFβ concentrations [42]. This point will be clarified in further studies by analyzing accumulation of other γ-secretase substrates, like CTF, p75, etc.

Aβ42-immunoreactivity was detected by single-neuron microdensitometry using the 6E10 and 4G8 monoclonal antibodies as probes. Since the peptide epitopes (AA 1–16) targeted by 6E10 and 4G8 are shared by the Aβ42 and the Aβ40 peptides as well as by the APP precursor and the sAPPβ and CTFβ cleavage by-products, these antibody can recognize all the above species [33]. Moreover, the possibility that abnormally trafficked APP or CTFβ (but not sAPPβ, which is extracellularly released) and other APP-derived peptides like C99 [43] may also contribute to the immunostaining observed in neurons from Tg2576 mice cannot be ruled out at present. Increasing attention is currently being paid to intraneuronal Aβ, generated *via* the endosomal system and accumulated in endosomes/lysosomes, mitochondria and autophagosomes, as an early player in the pathogenesis of AD [10]. In fact, intraneuronal Aβ accumulation occurs well before the increase of extracellular Aβ [10], it is detectable in plaque-free animals [22], and converging reports indicate a key pathogenetic role of intraneuronal β-amyloid in neurodegeneration and AD pathology [44,45]. For example, the intracellular concentration of Aβ42, the most toxic Aβ variant, in pyramidal (CA1) human neurons has been estimated to be 3 μM in the case of sporadic AD patients, as compared to 660 nM in the case of neurons from control, unaffected subjects [46]. Intraneuronal Aβ severely affects neurons viability [8,9,47] and vulnerability to various neurotoxic challenges [48]. Also, elevated levels of intraneuronal Aβ42 have been reported to be causally related to the activation of the protein kinases that are responsible for intracellular tau hyperphosphorylation in early stages of the disease [49,50]. Moreover, intraneuronal Aβ accumulation has been shown to play a key role in synapse loss and dysfunction [10,51]. CHF5074 was previously shown to restore the levels of synaptophysin [21], to reduce the accumulation of native hyperphosphorylated tau, and to decrease brain GSK-3β levels, a secondary effect presumably associated with the reduction of intraneuronal Aβ [52], thus suggesting an effect of this molecule on the most relevant molecular players involved in AD pathology.

In both CHF5074- and DAPT-treated animals there was a normalization of dendritic spine density in CA1/2 pyramidal neurons compared to vehicle-treated Tg2576 mice. With the use of two-photon confocal microscopy, DAPT has recently been shown to reduce dendritic spine density when administered acutely to wild-type mice [53]. It should be noted, however, that pharmacological modulation of wild-type and mutated-APP are quite different [54] and that the effect of APP on dendritic spine number is still highly controversial [55]. Moreover, multiple and diverse events, such as intracellular soluble or extracellular fibrillar Aβ levels, or the combination of intracellular soluble Aβ and hyperphosphorylated tau, can lead to dendritic spine loss [56]. Thus, amyloid clearance from neurons and from the brain by CHF5074 and DAPT may positively affect structural aspects of hippocampal circuits (e.g., dendritic spine density), but this is not enough, *per se*, to sustain cognitive performance, as suggested by the divergent effects of the two drugs on novel object recognition test.

Various dysfunctions, some of which precede cortical atrophy and amyloid plaque deposition, are causally associated with AD neurodegeneration. These include neuroinflammation, neurite abnormalities leading to dendritic spine loss, neuronal cell death and aberrant cell cycle events. Epidemiological analyses support the notion that intake of nonsteroidal anti-inflammatory drugs (NSAIDs) can reduce the risk and delay the onset of AD. In contrast to this positive preventive trend, therapeutic studies testing NSAID efficacy in AD patients have been largely unsuccessful so far [57]. A possible explanation for these disappointing, and apparently controversial results may be a wrong therapeutic time-window. In fact, while the occurrence of plaque-dependent inflammation in AD has been extensively documented in both human specimens and transgenic animal models of the disease, clinical and experimental evidence for the occurrence of inflammation in preclinical, asymptomatic phases of AD pathology are scanty [58]. In transgenic mouse models of AD, cerebrovascular inflammation has been reported to occur before plaque deposition [59] and various treatments targeting neuroinflammation have been proved to be effective on cognition maintenance or recovery [57]. Moreover, age-dependently enhanced neuroinflammatory processes may play an important role in neuronal death or dysfunction, possibly inducing spine pathology [56] as well as cell cycle alterations [30]. Thus, in the present study the possible anti-neuroinflammatory properties of CHF5074 and DAPT have also been assessed.

As revealed by the present results, while a substantial reduction of microglia activation was observed in CHF5074-treated animals, no effect on Iba1 immunostaining was detected following treatment with DAPT. Similar results with CHF5074 were obtained in a previous shorter-term study [21]. These data suggest that, at least under the present experimental conditions, CHF5074, but not DAPT, exerts an anti-neuroinflammatory activity. It should be mentioned that while a partial γ-secretase inactivation has a protective role on amyloid pathology and inflammation, the genetic ablation of presenilin causes progressive inflammatory responses [60]. Thus, the modulatory γ-secretase activity exerted by CHF5074 could positively affect initial microglia activation, while blockage of γ-secretase activity by DAPT is likely to be completely ineffective on this particular target. In line with this view, it has been reported recently that inhibition of lipopolysaccharide-induced γ-secretase activity by DAPT interferes with immune and anti-inflammatory regulatory pathways in the brain [61], while acute DAPT administration restraints microglia activation [62,63]. Recently, it has also been proposed that ibuprofen and its derivatives, including CHF5074, besides reducing Aβ pathology and neuroinflammation, modulate astrocyte reactivity through a Rho-GTPase/PAK/ERK-dependent signalling pathway [23]. In keeping with this purported mode of action, we have previously shown that chronic treatment of Tg2576 mice with CHF5074 causes astrocyte hypertrophy and their accumulation around large Aβ deposits [21] -another mechanism that may contribute to the neuroprotective and functional recovery effects promoted by this compound. This result was also confirmed in this study (Additional file 3: Figure S3). We also observed that DAPT also upregulates GFAP-IR but not Iba1, thus further suggesting that microglia activation is a property of CHF5074.

The anti-neuroinflammatory action produced by chronic treatment with CHF5074 may counteract cell death caused by oxidative damage. Ectopic expression of cell cycle proteins identifies neuronal populations undergoing neurodegeneration [63-65] and aberrant cell cycle events have been detected in Tg2576 mice starting from 6 months of age. Thus, the marked reduction of cyclin A-positive neurons (from 48% to 18%) elicited by CHF5074, but not DAPT, points to a fairly strong neuroprotective effect of the former compound. The mechanisms underlying cell-cycle re-entry and cell-cycle protein expression in AD neurons are still obscure [14,65]. It is worth noting, however, that alterations in brain microglia have been shown to occur concomitantly with the appearance of ectopic cell-cycle events and that NSAIDs prevent, but do not reverse, neuronal cell cycle re-entry in the R1.40 mouse model of AD [30]. The treatment regimen utilized in this study strongly supports this conclusion. However, microglia activation and neuroinflammation, are also triggered by intraneuronal Aβ accumulation at an early stage of the disease [66] and, conversely, inflammation negatively affects Aβ efflux from the brain [67]. Thus, the anti-inflammatory and cell cycle normalization properties of CHF5074 combined with its Aβ clearance-promoting effect may explain the superior neuroprotective action of this compound compared to DAPT.

## Conclusion

The present findings suggest that chronic treatment of Tg2576 mice with CHF5074, but not DAPT, effectively prevents AD symptoms (*e.g*., plaque accumulation and cognitive impairment) and positively affects various neurodegeneration-related events, some of which may occur earlier than, and be not directly linked to, massive Aβ accumulation. The most significant of the latter effects are a marked quenching of microglia activation and associated neuroinflammation as well as a reduced frequency of anomalous cell cycle re-entry events in affected neurons. Notably, the beneficial effects of CHF5074 remain well detectable till an extremely late age, after a one year-long chronic treatment. The multimodal action of CHF5074 appears to be in line with the increasingly recognized multifactorial nature of AD pathology, whose treatment can greatly benefit from the ability of candidate therapeutic compounds to act on more than one target [15,67]. Taken together, the results of this extensive comparative analysis suggest that a specific subset of effects not so closely related to Aβ accumulation and APP processing may play a crucial role in dictating the outcome of neurodegeneration and response to therapy. These less obvious effects, most notably the promotion of glia and neuronal cell cycle homeostasis, may represent key targets for the development and monitoring of second generation AD modifying drugs.

## Competing interest

This study was sponsored by Chiesi Farmaceutici, Parma, Italy. Bruno P. Imbimbo and Gino Villetti are employees of Chiesi Farmaceutici. The other authors declare no competing interest.

## Authors’ contribution

SS, CM and VB carried out immunohistochemical experiments and related quantification; LL carried out Golgi staining and spine quantification; AG carried out animal experiments; MG was responsable for animal care; MF carried out plasma assays; LF contributed to manuscript preparation; VP and MFB carried out plaque and microglia staining and quantification; GV was responsable for drugs PK and administration schema; ARV and SO performed Abeta oligomer analysis and contributed to manuscript preparation; LG, LC, and BPI have designed the study, performed statistical analysis and wrote the manuscript. All authors read and approved the final manuscript.

## Supplementary Material

Additional file 1: Figure S2 A, B: Representative images of activated microglia (IBA1 immunostaining) of CA1 hippocampal cortex of Tg2576 vehicle (A) and DAPT (B) treated animals. C, D: Representative images of 6E10 immunostaining of cerebral cortex of Tg2576 vehicle (C) and DAPT (D) treated animals. Click here for file

Additional file 2: Figure S1 Representative example of SDS-PAGE fractionation (4-12% Bis-Tris Midi gradient gel) and immunoblot analysis (primary antibody: 6E10 mAb, 1:500; secondary antibody: goat anti-mouse, IrDye 680-labeled antibody, 1:3000) carried out on “low-detergent”, 0.01% NP40/0.1% SDS brain extracts (enriched in extracellular Aβ). Immune-reactive bands were visualized by near-infrared fluorescence (Odyssey imager, LI-COR). Non-specific, 6E10 mAb cross-reactive polypeptides (*arrow*), present in both wild-type and Tg2576 brain extracts, were used as loading controls and internal references for data normalization. Synthetic prefibrillar Aβ42(n) prepared according to Lambert et al. [32], with *n*-values ranging from 1 to 4 (not shown), was used as size standard for electrophoretic analysis. Immune-reactive bands were quantified as “near-infrared fluorescence” (NIRF) arbitrary units (see ‘Methods’ for additional details). Click here for file

Additional file 3: Figure S3 Representative images of 6E10 (green) and GFAP- (red) immonostaining in the cerebral cortex of Tg2576 vehicle- (A) and DAPT 375ppm- (B) treated animals. A large number of plaques is observed in both groups, while the GFAP-immunostaining is strongly up-regulated in treated animal. C. semiquantitative evaluation of GFAP immunostaining in the experimental groups. The analysis was performed by evaluating the percentage of plaques surrounded by reactive astrocytes, as evaluated in an area doubling the plaque diameter (see Imbimbo et al.2010, for details). Treated animals show an intense upregulation of GFAP-immunostaining around the plaques. Statisical analysi: one-way ANOVA and Dunnet’s post-hoc test. * p<0.05; **p<0.001. Click here for file
