# Supplementary material for: Multi-target action of the novel anti-Alzheimer compound CHF5074: in vivo study of long term treatment in Tg2576 mice
Source: BMC Neurosci. 2013 Apr 5;14:44. doi: 10.1186/1471-2202-14-44 (PMC3626610; doi:10.1186/1471-2202-14-44)
Supplement: Additional file 1: Figure S2 — A, B: Representative images of activated microglia (IBA1 immunostaining) of CA1 hippocampal cortex of Tg2576 vehicle (A) and DAPT (B) treated animals. C, D: Representative images of 6E10 immunostaining of cerebral cortex of Tg2576 vehicle (C) and DAPT (D) treated animals. [file 1471-2202-14-44-S1.ppt]

## Slide 1
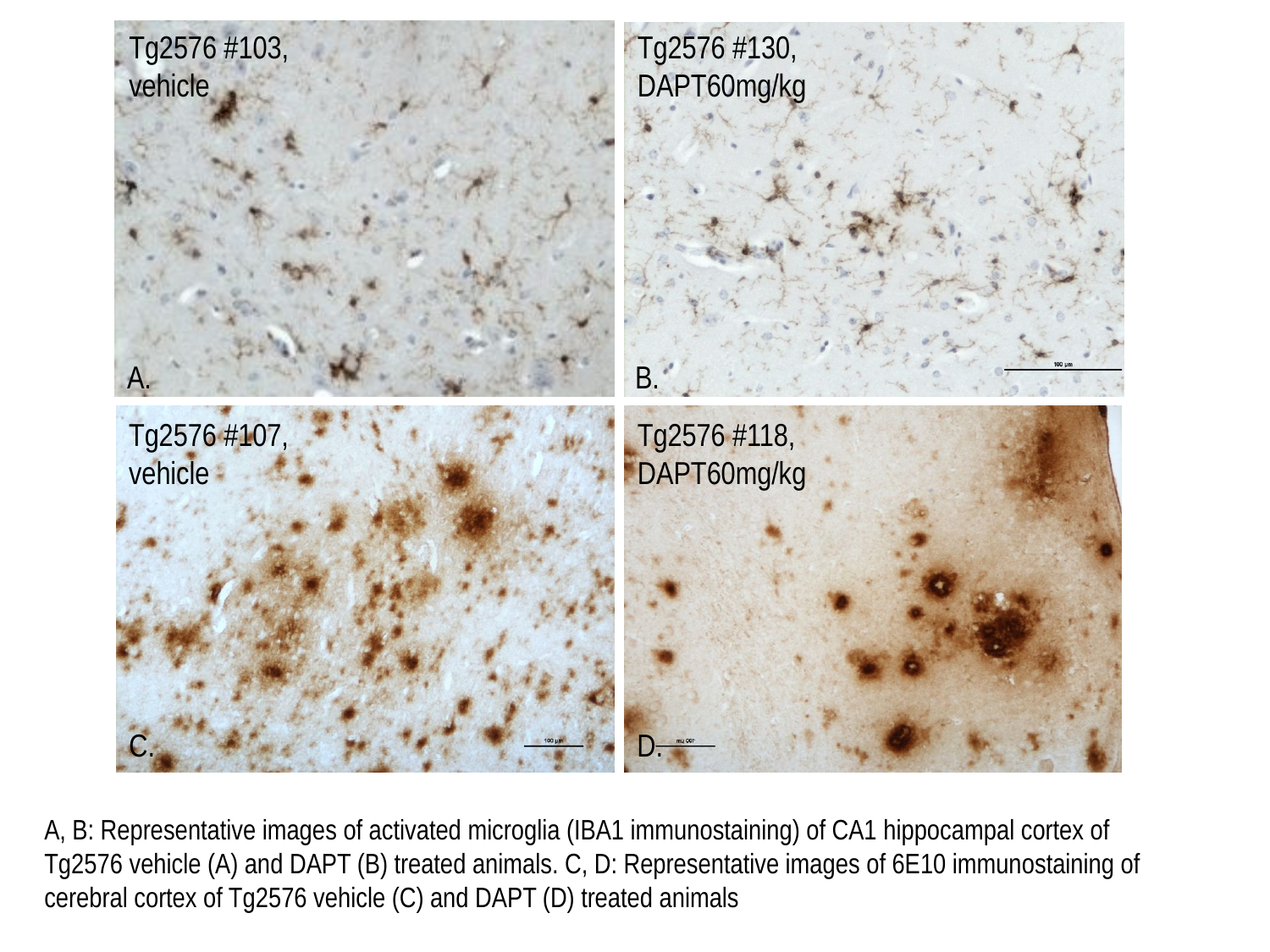

Tg2576 #103, vehicle
Tg2576 #130,
DAPT60mg/kg
A.				B.
Tg2576 #107, vehicle
Tg2576 #118,
DAPT60mg/kg
C.				D.
A, B: Representative images of activated microglia (IBA1 immunostaining) of CA1 hippocampal cortex of Tg2576 vehicle (A) and DAPT (B) treated animals. C, D: Representative images of 6E10 immunostaining of cerebral cortex of Tg2576 vehicle (C) and DAPT (D) treated animals
