# Supplementary material for: Multi-target action of the novel anti-Alzheimer compound CHF5074: in vivo study of long term treatment in Tg2576 mice
Source: BMC Neurosci. 2013 Apr 5;14:44. doi: 10.1186/1471-2202-14-44 (PMC3626610; doi:10.1186/1471-2202-14-44)
Supplement: Additional file 2: Figure S1 — Representative example of SDS-PAGE fractionation (4-12% Bis-Tris Midi gradient gel) and immunoblot analysis (primary antibody: 6E10 mAb, 1:500; secondary antibody: goat anti-mouse, IrDye 680-labeled antibody, 1:3000) carried out on “low-detergent”, 0.01% NP40/0.1% SDS brain extracts (enriched in extracellular Aβ). Immune-reactive bands were visualized by near-infrared fluorescence (Odyssey imager, LI-COR). Non-specific, 6E10 mAb cross-reactive polypeptides (arrow), present in both wild-type and Tg2576 brain extracts, were used as loading controls and internal references for data normalization. Synthetic prefibrillar Aβ42(n) prepared according to Lambert et al. [32], with n-values ranging from 1 to 4 (not shown), was used as size standard for electrophoretic analysis. Immune-reactive bands were quantified as “near-infrared fluorescence” (NIRF) arbitrary units (see ‘Methods’ for additional details). [file 1471-2202-14-44-S2.ppt]

## Slide 1
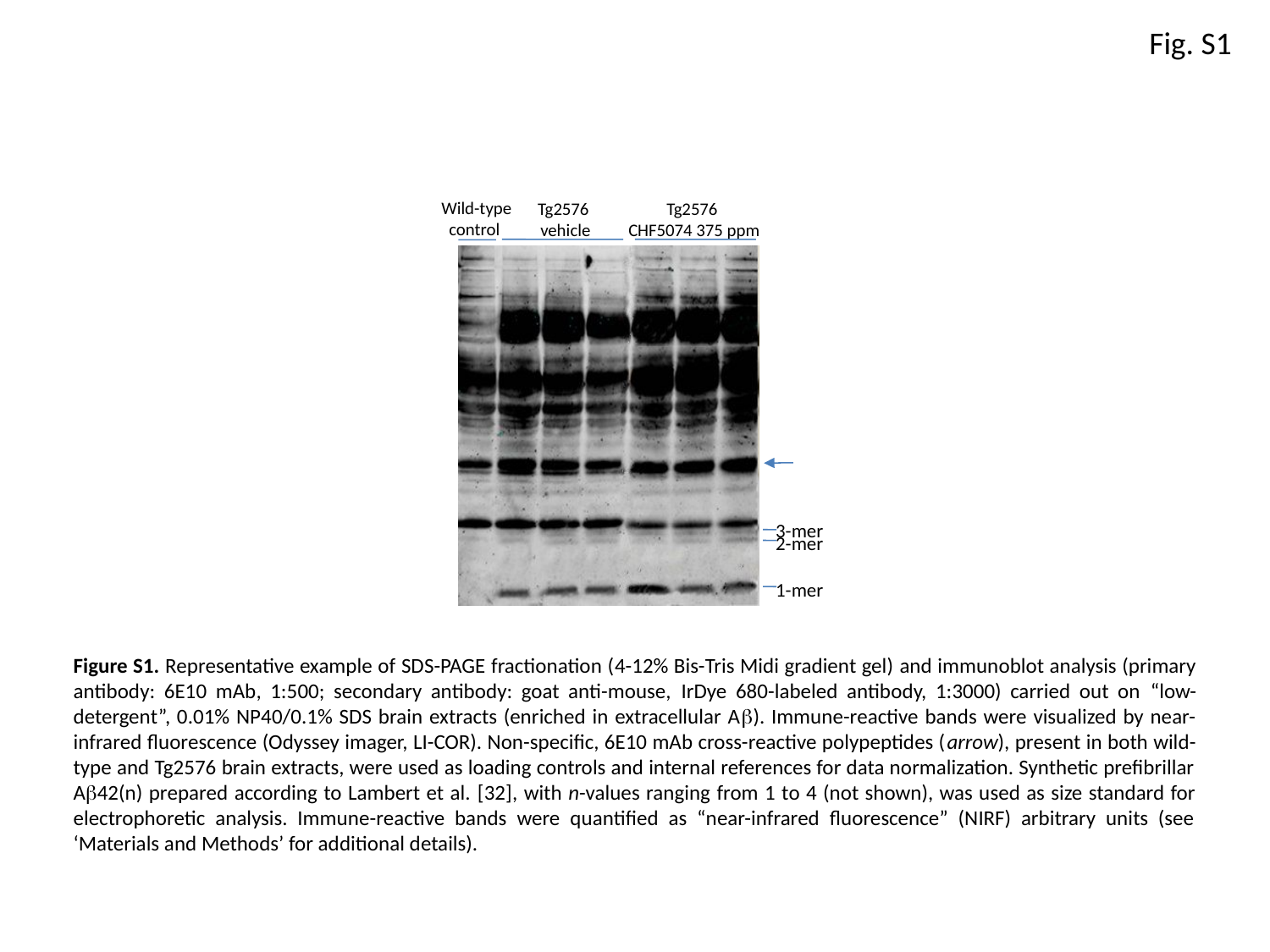

Fig. S1
Wild-type
control
Tg2576
vehicle
Tg2576
CHF5074 375 ppm
3-mer
2-mer
1-mer
Figure S1. Representative example of SDS-PAGE fractionation (4-12% Bis-Tris Midi gradient gel) and immunoblot analysis (primary antibody: 6E10 mAb, 1:500; secondary antibody: goat anti-mouse, IrDye 680-labeled antibody, 1:3000) carried out on “low-detergent”, 0.01% NP40/0.1% SDS brain extracts (enriched in extracellular Aβ). Immune-reactive bands were visualized by near-infrared fluorescence (Odyssey imager, LI-COR). Non-specific, 6E10 mAb cross-reactive polypeptides (arrow), present in both wild-type and Tg2576 brain extracts, were used as loading controls and internal references for data normalization. Synthetic prefibrillar Aβ42(n) prepared according to Lambert et al. [32], with n-values ranging from 1 to 4 (not shown), was used as size standard for electrophoretic analysis. Immune-reactive bands were quantified as “near-infrared fluorescence” (NIRF) arbitrary units (see ‘Materials and Methods’ for additional details).
