# Supplementary material for: Multi-target action of the novel anti-Alzheimer compound CHF5074: in vivo study of long term treatment in Tg2576 mice
Source: BMC Neurosci. 2013 Apr 5;14:44. doi: 10.1186/1471-2202-14-44 (PMC3626610; doi:10.1186/1471-2202-14-44)
Supplement: Additional file 3: Figure S3 — Representative images of 6E10 (green) and GFAP- (red) immonostaining in the cerebral cortex of Tg2576 vehicle- (A) and DAPT 375ppm- (B) treated animals. A large number of plaques is observed in both groups, while the GFAP-immunostaining is strongly up-regulated in treated animal. C. semiquantitative evaluation of GFAP immunostaining in the experimental groups. The analysis was performed by evaluating the percentage of plaques surrounded by reactive astrocytes, as evaluated in an area doubling the plaque diameter (see Imbimbo et al.2010, for details). Treated animals show an intense upregulation of GFAP-immunostaining around the plaques. Statisical analysi: one-way ANOVA and Dunnet’s post-hoc test. * p<0.05; **p<0.001. [file 1471-2202-14-44-S3.pdf]

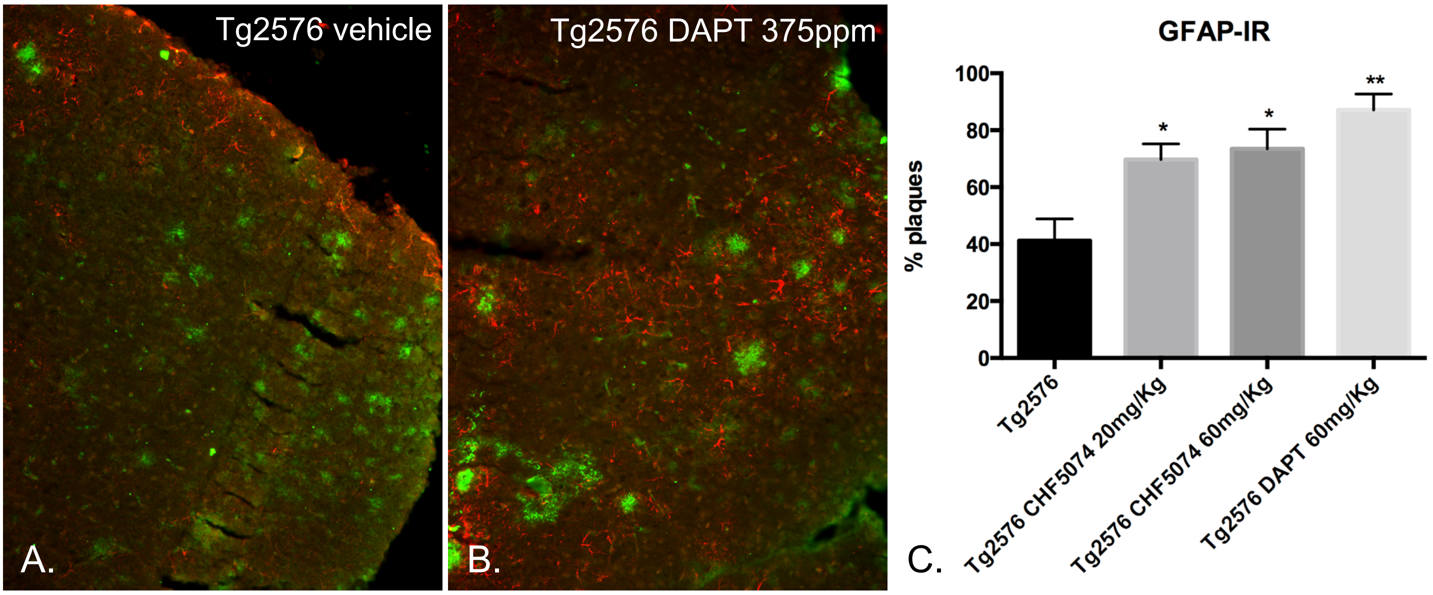

Supplementary Fig 3.

Representative images of 6E10 (green) and GFAP- (red) immonostaining in the cerebral cortex of Tg2576 vehicle- (A) and DAPT 375ppm- (B) treated animals. A large number of plaques is observed in both groups, while the GFAP-immunostaining is strongly up-regulated in treated animal. C. semiquantitative evaluation of GFAP immunostaining in the experimental groups. The analysis was performed by evaluating the percentage of plaques surrounded by reactive astrocytes, as evaluated in an area doubling the plaque diameter(see Imbimbo et al.2010, for details). Treated animals show an intense up-regulation of GFAP-immunostaining around the plaques. Statisical analysi: one-way ANOVA and Dunnet’s post-hoc test. * $p<0.05$; ** $p<0.001$.
